# Combined Hepatocellular-Cholangiocarcinoma: What the Multidisciplinary Team Should Know

**DOI:** 10.3390/diagnostics12040890

**Published:** 2022-04-02

**Authors:** Carmen Cutolo, Federica Dell’Aversana, Roberta Fusco, Giulia Grazzini, Giuditta Chiti, Igino Simonetti, Federico Bruno, Pierpaolo Palumbo, Luca Pierpaoli, Tommaso Valeri, Francesco Izzo, Andrea Giovagnoni, Roberto Grassi, Vittorio Miele, Antonio Barile, Vincenza Granata

**Affiliations:** 1 Department of Medicine, Surgery and Dentistry, University of Salerno, 84081 Salerno, Italy; carmencutolo@hotmail.it; 2 Division of Radiology, Università degli Studi della Campania Luigi Vanvitelli, 80138 Naples, Italy; federica.dellaversana@unicampania.it (F.D.); roberto.grassi@unicampania.it (R.G.); 3 Medical Oncology Division, Igea SpA, 80013 Napoli, Italy; 4 Department of Radiology, Azienda Ospedaliero-Universitaria Careggi, 50134 Florence, Italy; grazzini.giulia@gmail.com (G.G.); giudittachiti@gmail.com (G.C.); vmiele@sirm.org (V.M.); 5 Italian Society of Medical and Interventional Radiology (SIRM), SIRM Foundation, Via della Signora 2, 20122 Milan, Italy; federico.bruno.1988@gmail.com (F.B.); palumbopierpaolo89@gmail.com (P.P.); a.giovagnoni@univpm.it (A.G.); antonio.barile@univaq.it (A.B.); 6 Division of Radiology, Istituto Nazionale Tumori IRCCS Fondazione Pascale–IRCCS di Napoli, 80131 Naples, Italy; i.simonetti@istitutotumori.na.it (I.S.); v.granata@istitutotumori.na.it (V.G.); 7 Department of Applied Clinical Sciences and Biotechnology, University of L’Aquila, 67100 L’Aquila, Italy; 8 Department of Diagnostic Imaging, Area of Cardiovascular and Interventional Imaging, Abruzzo Health Unit 1, 67100 L’Aquila, Italy; 9 Department of Clinical, Special and Dental Sciences, Marche Polytechnic University, 60126 Ancona, Italy; l.pierpaoli@univpm.it (L.P.); t.valeri@univpm.it (T.V.); 10 Division of Hepatobiliary Surgical Oncology, Istituto Nazionale Tumori IRCCS Fondazione Pascale–IRCCS di Napoli, 80131 Naples, Italy; f.izzo@istitutotumori.na.it

**Keywords:** cHCC-CCA, diagnosis, MRI, CT, LI-RADS, surgical resection, ablative treatment

## Abstract

Combined hepatocellular-cholangiocarcinoma (cHCC-CCA) is a rare type of primary liver malignancy. Among the risk factors, hepatitis B and hepatitis C virus infections, cirrhosis, and male gender are widely reported. The clinical appearance of cHCC-CCA is similar to that of HCC and iCCA and it is usually silent until advanced states, causing a delay of diagnosis. Diagnosis is mainly based on histology from biopsies or surgical specimens. Correct pre-surgical diagnosis during imaging studies is very problematic and is due to the heterogeneous characteristics of the lesion in imaging, with overlapping features of HCC and CCA. The predominant histological subtype within the lesion establishes the predominant imaging findings. Therefore, in this scenario, the radiological findings characteristic of HCC show an overlap with those of CCA. Since cHCC-CCAs are prevalent in patients at high risk of HCC and there is a risk that these may mimic HCC, it is currently difficult to see a non-invasive diagnosis of HCC. Surgery is the only curative treatment of HCC-CCA. The role of liver transplantation (LT) in the treatment of cHCC-CCA remains controversial, as is the role of ablative or systemic therapies in the treatment of this tumour. These lesions still remain challenging, both in diagnosis and in the treatment phase. Therefore, a pre-treatment imaging diagnosis is essential, as well as the identification of prognostic factors that could stratify the risk of recurrence and the most adequate therapy according to patient characteristics.

## 1. Introduction

Combined hepatocellular-cholangiocarcinoma (cHCC-CCA) is an unusual subtype of primary liver malignancy, with an incidence that varies among different reports between 0.4 and 14.2% of all primitive hepatic cancers [1,2,3,4,5]. In the literature, it has been known by several terminologies, including mixed hepatocellular carcinomacholangiocarcinoma (HCC-CC), hybrid HCC-CC, or combined liver and bile duct carcinoma [6,7].

Among the risk factors, hepatitis B and hepatitis C virus infections, cirrhosis, and male gender are widely reported [8,9,10,11,12,13,14,15,16]. cHCC-CC is usually thought of as an aggressive tumour mainly due to its poor prognosis. This feature is also correlated to an erratum diagnosis during pre-surgical radiological studies, when the lesion is classified as an hepatocellular carcinoma (HCC) or intrahepatic mass-forming cholangiocarcinoma (iCCA) [17,18,19,20,21,22,23].

This study is a narrative review of the current knowledge of clinical features, pathology assessment, diagnosis, and treatment of cHCC-CCA.

## 2. Pathology Assessment

Histologically, cHCC-CCA is characterised by 2 distinct morphological patterns: HCC and intrahepatic CCA (iCCA) [24,25,26,27,28].

HCC-CCA’s first classification was developed by Allen and Lisa in 1949 [29]. They classified HCC-CC into 3 types:

- Type A: HCC and CCA are present at different sites in the same liver;
- Type B: HCC and iCCA are present at adjacent sites;
- Type C: HCC and iCCA are combined within the same tumour.

In 1985, Goodman et al., in a review of 24 cases of this tumour, described three different histologic types [30]. Type I or “collision tumour” was characterized by the presence of both HCC and iCCA in the same patient. Type II or “transitional tumour” was characterized by the presence of the same lesions in an area with intermediate differentiation between HCC and iCCA. Type III or “fibrolamellar tumour” resembled the HCC fibrolamellar, also containing mucin-producing pseudoglands. Type III differs from the others since it is typical in younger patients, who do not have cirrhosis [26].

The World Health Organization (WHO) introduced in 2010 the last classification system, which divides cHCC-CCA into two types: classical type (a single tumour with both differentiations) and cHCC-CC with stem cell features [31]. This last one can be sub-classified into three variants: “typical”, “intermediate cell”, and “cholangiolocellular” subtypes. Recently, it has been demonstrated that the “stem cell” subtype can be proven in different types of hepatic tumours [32,33,34,35].

All primary liver cancer subtypes can be combined in the same nodule, and when it occurs, a precise description and percentage of each cancer type present in surgical specimens are recommended [36,37].

## 3. Epidemiology, Clinical Features, and Risks Factors

Nowadays, there is no clear profile of the patients affected by this rare primary hepatic neoplasm, being highly dependent on the geographic region. Indeed, the etiology and risk factors for this cancer may differ between different regions in the East and West. This reflects the different prevalence of risk factors in different countries. One-quarter of the global population is estimated to have non-alcoholic fatty liver disease (NAFLD). NAFLD is already the fastest-growing cause of HCC in the USA, France, and the UK. Globally, the prevalence of NAFLD-related HCC is likely to increase concomitantly with the growing obesity epidemic. The estimated annual incidence of HCC ranges from 0.5% to 2.6% among patients with NASH cirrhosis. The incidence of HCC among patients with non-cirrhotic NAFLD is lower, approximately 0.1 to 1.3 per 1000 patient-years. Although the incidence of NAFLD-related HCC is lower than that of HCC of other aetiologies, such as hepatitis C, more people have NAFLD than other liver diseases [38]. Chronic liver damage and subsequent liver cirrhosis are strong oncogenic factors [39]. Several studies have highlighted risk factors such as male gender, cirrhosis, hepatitis infection, family history of liver cancer, heavy alcohol consumption, and diabetes mellitus [40,41]. In Asian studies, the high man: woman ratio and prevalence of virus B infection in cHCC-CCA patients are similar to HCC patients compared to iCC patients [40,41,42]. Otherwise, in Western reports, there is a lesser male predominance and a lower prevalence of virus B infection compared to virus C infection [43]. Furthermore, several researchers have described that cHCC-CCA has a poor prognosis and more aggressive behavior compared to HCC and/or iCCA alone [44,45].

The clinical appearance of cHCC-CCA is analogous to that of HCC and iCCA. In fact, it has usually nonspecific symptoms characterized by fatigue, abdominal pain, weight loss, pruritus, and, in advanced states, jaundice, ascites, acute cholangitis, and hepatomegaly [46]. Additionally, in cHCC-CCA patients, it is possible to find several tumour markers, such as alpha-fetoprotein, carcinoembryonic antigen, and carbohydrate antigen 19.9 [47,48,49]. Although these biomarkers are not specific to cHCC-CCA and may be found even in non-oncological disorders, an increase in their levels should be investigated [50].

## 4. Diagnosis

Diagnosis of cHCC-CCA is mainly based on histology from biopsies or surgical specimens [51,52]. Liver biopsy had an estimated sensitivity of 48% and specificity of 100% for the diagnosis of cHCC-CCA in the pre-surgical setting [53].

**Figure 1:**
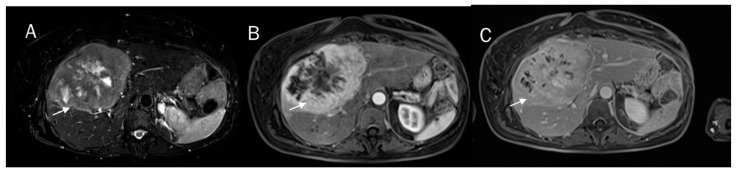
cHCC-CCA on IV-VIII hepatic segment. The lesion (arrow) shows the hyperintense signal on T2-W sequences (**A**) and progressive contrast enhancement during arterial (**B**) and venous (**C**) phases of contrast studies, features typical of iCCA.

**Figure 2:**
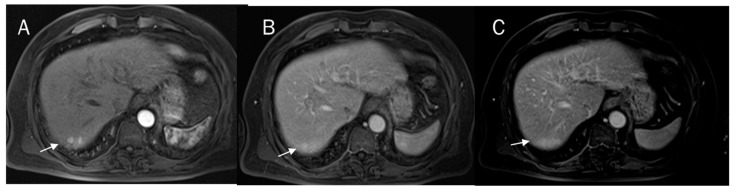
Satellite nodules (arrows) on VII hepatic segment with progressive contrast enhancement during the arterial (**A**), portal (**B**), and late (**C**) phases of contrast study.

**Figure 3:**
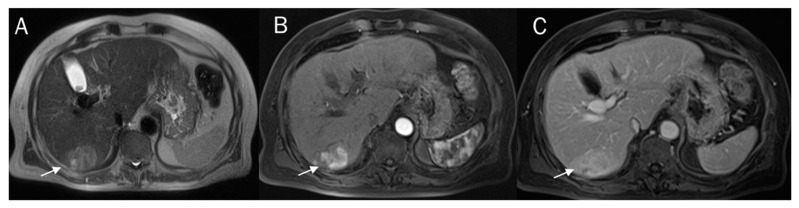
cHCC-CCA on VI hepatic segment. The lesion (arrows) shows hyperintense signal on T2-W sequences (**A**) and progressive contrast enhancement during the arterial (**B**) and venous (**C**) phases of contrast studies.

A correct pre-surgical diagnosis during imaging studies is very problematic due to the heterogeneous characteristics of the lesion in imaging, with overlapping features of HCC and CCA (Figure 1) [54,55,56,57,58,59,60,61,62,63,64,65]. The predominant histological subtype within the lesion establishes the predominant imaging findings. Therefore, in this scenario, the radiological findings characteristic of HCC show an overlap with those of CCA. Since cHCC-CCAs are prevalent in patients with high-risk HCC and there is the risk that these may mimic HCC, it is difficult currently to see a non-invasive diagnosis of HCC [66,67,68,69,70]. Current imaging-based criteria for HCC diagnosis have been grouped into Liver Imaging Reporting and Data System (LI-RADS), which is a scheme for interpreting and reporting imaging features in computed tomography (CT) and magnetic resonance (MR) studies in patients at risk of HCC [71,72,73,74,75,76]. In LI-RADS, key imaging features and ancillary features are evaluated, and diagnosis is due to the presence of the major features that are used to classify LI-RADS-category 3 (LR-3), LI-RADS-category 4 (LR-4), and LI-RADS-category 5 (LR-5), including arterial phase hyper potentiation, tumour diameter, washout, capsule appearance, and threshold growth. Ancillary features that aid in the HCC diagnosis include lesion signal hypointensity during the hepatobiliary phase of an MR contrast study with a liver-specific contrast agent, transitional phase hypointensity, mild to moderate signal hyperintensity in T2-W sequences, restricted diffusion, mosaic architecture, nodule architecture in the nodule, intralesional fat, lesional iron, or fat sparing, blood products, and a diameter increase that is less than the growth threshold [77,78,79,80,81]. Although vascular criteria are the main features that allow an accurate diagnosis, other data, such as restricted diffusion and the signal of T2-W sequences, could favor the characterization of the lesion. However, in the assessment of cHCC-CCA, when the predominant subtype is HCC, these data do not allow a proper diagnosis. On the other hand, for lesions with a predominant CCA component, characteristics that favor the diagnosis of LR-M category and the presence of satellite nodules (Figure 2) include hyperintense signal on T2-W, restricted diffusion, and the absence of capsule appearance in a nodule with peripheral and progressive contrast (Figure 3), should aid in the diagnosis of cHCC-CCA [77]. In addition, Granata et al. compared a control group of patients with a histological diagnosis of iCCA with cHCC-CCA and showed that T1 and T2-W signal intensity (SI), restricted diffusion, and transitional phase (TP) and hepatobiliary phase (HP) appearances allowed differentiation between mass-forming and mimicking ICCs with statistical significance, making MRI a valuable diagnostic tool for these lesions [7]. 

Considering the diagnostic criteria, the imaging modalities that should be chosen in cHCC-CCA assessment are CT and MRI. 

A protocol CT study should comprise multiphase imaging with thin collimation, including non-contrast, arterial, portal or venous, and delayed phase images. The introduction of new techniques such as dual-energy CT (DECT) and perfusion CT [82,83,84,85,86,87,88,89,90,91] has increased the diagnostic performance of CT in the characterization of focal liver lesions [92,93].

MRI studies can be performed with a 1.5 T or 3 T system using an abdominal-phased array coil. An MRI liver study protocol should comprise multi-shot fast spin-echo T2-weighted (W) images, with and without fat suppression; T1-W with double-echo chemical shift; T1-W before and after contrast administration, including arterial, portal and late-phase; and Diffusion sequences. When employing a hepatospecific contrast agent, hepatobiliary phase imaging can also be performed [94].

Today, MRI is unique compared to CT and US since it allows only one study protocol to assess conventional data obtained by T2-W and T1-W sequences, with functional data obtained by DWI, DCE-MRI, and Blood sequences. In this scenario, MRI is not only problem-solving but the first tool that should be used in an oncological setting [95,96,97,98,99,100,101,102,103,104,105,106,107,108,109,110,111,112,113,114,115,116,117,118,119,120,121,122,123,124,125]. However, imaging shows low diagnostic performance on its own, with a sensitivity of only 48% and a specificity of 81%, although the combination of imaging and biopsy can improve sensitivity (60%) and specificity (82%) [126]. Moreover, imaging is critical to guide liver biopsy and perform tumour staging [127].

## 5. Treatment

### 5.1. Surgery

**Figure 4:**
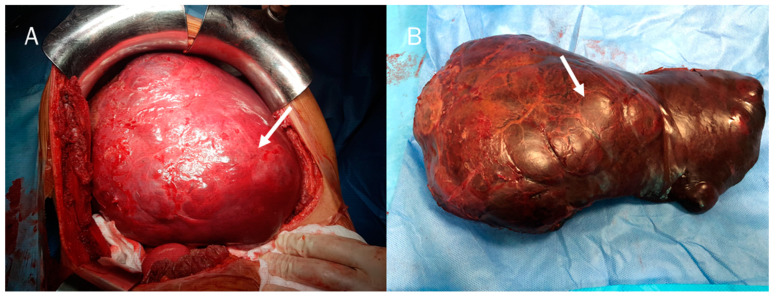
(**A**) cHCC-CCA during surgical resection (arrow); and (**B**) in surgical specimen (arrow).

**Figure 5:**
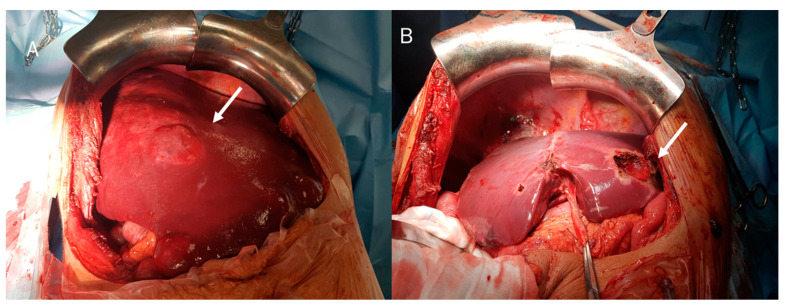
(**A**) cHCC-CCA on II hepatic segment during surgical resection (arrow); and (**B**) post-surgical resection features (arrow).

Surgery is the only curative treatment for cHCC-CCA (Figure 4 and Figure 5) [128]. According to current principles for oncologic liver surgery, liver resection aims to remove the lesion with adequate margins and with sufficient liver remnant volume. This needs a multi-parametric patient assessment for a correct evaluation of the lesion and functional liver status [129]. As demonstrated by the study of Ma et al., resection margin >10 mm has been correlated with protracted disease-free survival, since these resection margins allow eradication of satellite nodules and micro-tumours located in the same hepatic segment [130]. Several features could guide the surgical procedure, comprising liver functional status, patient general status, tumour size, localization, and vascular infiltration. As reported by Garancini et al. [131], aggressive surgical approaches were significantly correlated with prolonged survival with respect to non-surgical treatments (*p* < 0.001). Moreover, major hepatectomy for cHCC-CCA was associated with higher 5-year Overall Survival (OS) and Disease-Free Survival (DFS) rates with respect to minor hepatectomy [129]. Bearing in mind that the cHCC-CCA patient is a patient with cirrhosis, the degree of portal hypertension should be evaluated in surgical management since significant portal hypertension is an absolute contraindication to the main approaches [132]. Furthermore, the lymphatic pattern of tumour spread in cHCC-CCA requires routine hilar lymphadenectomy [133]. Also, transitional cHCC-CCA tends to infiltrate portal and hepatic veins as HCC and tends to invade lymph nodes as iCCA. Several studies [134,135] recommended liver resection with regional lymph node dissection to obtain oncological radicality in patients with transitional cHCC-CCA. However, it has not been demonstrated if lymphadenectomy improves the prognosis [136,137,138]. The need for routine lymphadenectomy should currently restrict the use of a laparoscopic approach only to centres with extensive expertise both in liver surgery and laparoscopy [139,140].

### 5.2. Liver Transplantation

The role of liver transplantation (LT) in the treatment of cHCC-CCA remains controversial. As with iCCAs, cHCC-CCA is a contraindication to liver transplantation due to historically high recurrence rates and poor OS. [141]. Several retrospective studies have assessed outcome for cHCC-CCA patients subjected to LT, reporting a recurrence rate of 40% [9,142,143,144]. However, due to the sample size analysed, the recurrence risk status post-LT remains problematic to evaluate. A systematic review reported a median DFS of 14.2 months and a median OS of 37.1 months [144]. Sapisochin et al. [142], in their multicenter matched cohort analysis, identified 42 iCCA (15 with cHCC-CCA) patients over a 10-year period. Researchers compared these patients to within-Milan criteria, HCC-matched controls, showing similar 5-year OS (78% vs. 86%) and recurrence risk (7% vs. 4%). However, these results correlate to the sample size assessed and lesser-advanced disease on the explant liver as compared to other published results. In addition, surveillance for this group was short, so data on disease stability over time are not available.

Lunsford et al. [145] conducted a propensity-matched analysis, within liver transplant recipients diagnosed with cHCC-CCA at explant (n = 12) were matched by pre- and post-transplant tumour characteristics 1:3 to patients with HCC (n = 36). cHCC-CCA tumours were more likely to be poorly differentiated and of a higher grade. When matched by pre-transplant characteristics, OS and RFS were inferior for cHCC-CCA, but the results were not statistically significant. When patients were compared by explant pathological criteria (diameter, differentiation, grade, and vascular invasion), recurrence rates remained minimally elevated for cHCC-CCA, but OS and RFS equalized (42% vs. 48% and 42% vs. 44%, at five years, respectively) [145]. All recurrences were found in patients with poorly differentiated lesions, with no patients having well- or moderately-differentiated tumours. These data favour the thesis that well- or moderately-differentiated cHCC-CCA patients could take advantage of LT. 

### 5.3. Locoregional Treatments

Few studies have analysed the effectiveness of transarterial chemoembolization (TACE) on cHCC-CCA. Kim et al. [146], from 1997 to 2009, recruited 50 patients with non-resectable cHCC-CCA. TACE produced a partial response or stable disease in 70% of patients, essentially in tumours with APHE (feature typical of HCC predominant subtype), with a median OS of 12.3 months. Better outcomes were reported in patients treated with TACE for recurrence after the surgical approach [147,148]. cHCC-CCA with global enhancement showed a significantly better response rate (complete remission + partial response) than the rim enhancement of the cHCC-CCA group (36% vs. 0%, *p* = 0.005), and it was comparable to that of the HCC-control group (35.6%, *p* = 0.97).

Data on radio-embolization (selective internal radiation therapy (SIRT)) and chemotherapy for non-resectable iCCA show that 22% of patients can be downstaged for surgical resection [149]. In the study by Malone et al., SIRT was associated with a 55% radiological response rate (15% complete response, 40% partial response), a 65% disease control rate, and a median OS of 9.3 months in 21 patients, suggesting a possible role for SIRT in locoregional control of cHCC-CCA [150]. However, since little data are currently available in the literature, more studies are necessary to establish the correct role of ablation treatments in cHCC-CCA patients. 

### 5.4. Systemic Treatments

Today, the data reported on cHCC-CCA patients unfit for surgery are restricted to retrospective analyses, evaluating the first-line therapies authorised for HCC (sorafenib) and iCCA (gemcitabine/platinum regimens) [151,152,153,154,155,156].

Kobayashi et al., in a multicentre retrospective analysis of systemic chemotherapy for unresectable cHCC-CCA, enrolled 36 patients: 12 patients underwent first-line chemotherapy consisting of gemcitabine/cisplatin, 11 with fluorouracil/cisplatin, 5 with sorafenib and 8 with others. A multivariate evaluation showed that the OS in the sorafenib monotherapy group was poor with respect to the platinum-containing regimens group. The authors concluded that the platinum-containing regimen had more favorable outcomes than the sorafenib treatment [153]. Salimon et al., in a multicentric study that included 30 patients with unresectable cHCC-CCA, showed that gemcitabine plus platinum is effective as a first-line treatment of advanced cHCC-CCA [154]. Similar results were found in a monocentric study of 68 patients with unresectable cHCC-CCA [155]. In a recent retrospective analysis of 99 cHCC-CCA patients, researchers compared a group of patients (n = 67) who received sorafenib with those who received cytotoxic chemotherapy. Among the two groups (sorafenib vs. cytotoxic chemotherapy), outcomes were not significantly different (ORR, 9.7% vs. 21.6%, *p* = 0.14; median PFS, 4.2 vs. 2.9 months, *p* = 0.52; median OS, 10.7 vs. 10.6 months, *p* = 0.34) [156]. 

A better understanding of the molecular basis of cancer would help develop targeted therapeutic agents against the druggable genetic aberrations identified in cancer genomes [157,158,159]. Tyrosine kinase inhibitors (TKIs) that target anaplastic lymphoma kinase (ALK) are particularly effective in the treatment of a distinct subset of lung adenocarcinoma carrying ALK fusions. FIG- ROS1, the first identified targetable fusion kinase in CC, has so far been reported in two patients. Very recently, a novel kinase fusion, FGFR2-BICC1, was detected in two CC cases [157]. FGFR2 fusions occur in 13.6% of intrahepatic cholangiocarcinoma cases. The expression pattern of these fusions in association with sensitivity to FGFR inhibitors warrants a new molecular classification of cholangiocarcinoma and suggests a new therapeutic approach to the disease [157]. 

### 5.5. Conclusions

Although cHCC-CCA represents a rare entity, this tumour remains challenging both in diagnosis and treatment. Therefore, a pre-treatment imaging diagnosis is essential, as well as the identification of prognostic factors that could stratify the recurrence risk and the most adequate therapy according to patient characteristics.

## Data Availability

All data are reported in this manuscript.

## References

[1] Jarnagin W.R., Weber S., Tickoo S.K., Koea J.B., Obiekwe S., Fong Y., DeMatteo R.P., Blumgart L.H., Klimstra D. (2002). Combined hepatocellular and cholangiocarcinoma: Demographic, clinical, and prognostic factors. Cancer.

[2] Lee W.-S., Lee K.-W., Heo J.-S., Kim S.-J., Choi S.-H., Kim Y.-I., Joh J.-W. (2006). Comparison of Combined Hepatocellular and Cholangiocarcinoma with Hepatocellular Carcinoma and Intrahepatic Cholangiocarcinoma. Surg. Today.

[3] Koh K.C., Lee H., Choi M.S., Lee J.H., Paik S.W., Yoo B.C., Rhee J.C., Cho J.W., Park C.K., Kim H.J. (2005). Clinicopathologic features and prognosis of combined hepatocellular cholangiocarcinoma. Am. J. Surg..

[4] Shin N., Choi J.A., Choi J.M., Cho E.-S., Kim J.H., Chung J.-J., Yu J.-S. (2020). Sclerotic changes of cavernous hemangioma in the cirrhotic liver: Long-term follow-up using dynamic contrast-enhanced computed tomography. Radiol. Med..

[5] De Filippo M., Puglisi S., D’Amuri F., Gentili F., Paladini I., Carrafiello G., Maestroni U., Del Rio P., Ziglioli F., Pagnini F. (2021). CT-guided percutaneous drainage of abdominopelvic collections: A pictorial essay. Radiol. Med..

[6] Blatchford F.W. (1952). Primary carcinoma of the liver; a critical analysis of sixteen cases. Gastroenterology.

[7] Granata V., Grassi R., Fusco R., Setola S.V., Belli A., Ottaiano A., Nasti G., La Porta M., Danti G., Cappabianca S. (2021). Intrahepatic cholangiocarcinoma and its differential diagnosis at MRI: How radiologist should assess MR features. Radiol. Med..

[8] Okuda K. (1997). Hepatocellular carcinoma: Clinicopathological aspects. J. Gastroenterol. Hepatol..

[9] Elshamy M., Presser N., Hammad A.Y., Firl D.J., Coppa C., Fung J., Aucejo F.N. (2017). Liver transplantation in patients with incidental hepatocellular carcinoma/cholangiocarcinoma and intrahepatic cholangiocarcinoma: A single-center experience. Hepatobiliary Pancreat. Dis. Int..

[10] Barabino M., Gurgitano M., Fochesato C., Angileri S.A., Franceschelli G., Santambrogio R., Mariani N.M., Opocher E., Carrafiello G. (2021). LI-RADS to categorize liver nodules in patients at risk of HCC: Tool or a gadget in daily practice?. Radiol. Med..

[11] Cholangiocarcinoma Working Group (2020). Italian Clinical Practice Guidelines on Cholangiocarcinoma—Part I: Classification, diagnosis and staging. Dig. Liver Dis..

[12] Cholangiocarcinoma Working Group (2020). Italian Clinical Practice Guidelines on Cholangiocarcinoma—Part II: Treatment. Dig. Liver Dis..

[13] Gabelloni M., Di Nasso M., Morganti R., Faggioni L., Masi G., Falcone A., Neri E. (2020). Application of the ESR iGuide clinical decision support system to the imaging pathway of patients with hepatocellular carcinoma and cholangiocarcinoma: Preliminary findings. Radiol. Med..

[14] Granata V., Bicchierai G., Fusco R., Cozzi D., Grazzini G., Danti G., De Muzio F., Maggialetti N., Smorchkova O., D’Elia M. (2021). Diagnostic protocols in oncology: Workup and treatment planning. Part 2: Abbreviated MR protocol. Eur. Rev. Med. Pharmacol. Sci..

[15] Gatti M., Calandri M., Bergamasco L., Darvizeh F., Grazioli L., Inchingolo R., Ippolito D., Rousset S., Veltri A., Fonio P. (2020). Characterization of the arterial enhancement pattern of focal liver lesions by multiple arterial phase magnetic resonance imaging: Comparison between hepatocellular carcinoma and focal nodular hyperplasia. Radiol. Med..

[16] Orlacchio A., Chegai F., Roma S., Merolla S., Bosa A., Francioso S. (2020). Degradable starch microspheres transarterial chemoembolization (DSMs-TACE) in patients with unresectable hepatocellular carcinoma (HCC): Long-term results from a single-center 137-patient cohort prospective study. Radiol. Med..

[17] Stavraka C., Rush H., Ross P. (2018). Combined hepatocellular cholangiocarcinoma (cHCC-CC): An update of genetics, molecularbiology, and therapeutic interventions. J. Hepatocell Carcinoma.

[18] Argalia G., Tarantino G., Ventura C., Campioni D., Tagliati C., Guardati P., Kostandini A., Marzioni M., Giuseppetti G.M., Giovagnoni A. (2021). Shear wave elastography and transient elastography in HCV patients after direct-acting antivirals. Radiol. Med..

[19] Rampado O., Depaoli A., Marchisio F., Gatti M., Racine D., Ruggeri V., Ruggirello I., Darvizeh F., Fonio P., Ropolo R. (2021). Effects of different levels of CT iterative reconstruction on low-contrast detectability and radiation dose in patients of different sizes: An anthropomorphic phantom study. Radiol. Med..

[20] Ponnoprat D., Inkeaw P., Chaijaruwanich J., Traisathit P., Sripan P., Inmutto N., Na Chiangmai W., Pongnikorn D., Chitapanarux I. (2020). Classification of hepatocellular carcinoma and intrahepatic cholangiocarcinoma based on multi-phase CT scans. Med. Biol. Eng. Comput..

[21] Tsunematsu S., Chuma M., Kamiyama T., Miyamoto N., Yabusaki S., Hatanaka K., Mitsuhashi T., Kamachi H., Yokoo H., Kakisaka T. (2015). Intratumoral artery on contrast-enhanced computed tomography imaging: Differentiating intrahepatic cholangiocarcinoma from poorly differentiated hepatocellular carcinoma. Gastrointest. Radiol..

[22] Schicchi N., Fogante M., Palumbo P., Agliata G., Pirani P.E., Di Cesare E., Giovagnoni A. (2020). The sub-millisievert era in CTCA: The technical basis of the new radiation dose approach. Radiol. Med..

[23] Nakamura Y., Higaki T., Honda Y., Tatsugami F., Tani C., Fukumoto W., Narita K., Kondo S., Akagi M., Awai K. (2021). Advanced CT techniques for assessing hepatocellular carcinoma. Radiol. Med..

[24] Joo I., Kim H., Lee J.M. (2015). Cancer Stem Cells in Primary Liver Cancers: Pathological Concepts and Imaging Findings. Korean J. Radiol..

[25] Gera S., Ettel M., Acosta-Gonzalez G., Xu R. (2017). Clinical features, histology, and histogenesis of combined hepatocellular-cholangiocarcinoma. World J. Hepatol..

[26] Craig J.R., Peters R.L., Edmondson H.A., Omata M. (1980). Fibrolamellar carcinoma of the liver: A tumor of adolescents and young adults with distinctive clinico-pathologic features. Cancer.

[27] Mirabile A., Lucarelli N.M., Sollazzo E.P., Stabile Ianora A.A., Sardaro A., Mirabile G., Lorusso F., Racanelli V., Maggialetti N., Scardapane A. (2021). CT pulmonary angiography appropriateness in a single emergency department: Does the use of revised Geneva score matter?. Radiol. Med..

[28] Mathew R.P., Sam M., Raubenheimer M., Patel V., Low G. (2020). Hepatic hemangiomas: The various imaging avatars and its mimickers. Radiol. Med..

[29] Allen R.A., Lisa J.R. (1949). Combined liver cell aNd bile duct carcinoma. Am. J. Pathol..

[30] Goodman Z.D., Ishak K.G., Langloss J.M., Sesterhenn I.A., Rabin L. (1985). Combined hepatocellular-cholangiocarcinoma. A histologic and immunohistochemical study. Cancer.

[31] Bosman F.T., Carneiro F., Hruban R.H., Theise N.D. (2010). WHO Classification of Tumours of the Digestive System.

[32] Gentile D., Donadon M., Di Tommaso L., Samà L., Franchi E., Costa G., Lleo A., Torzilli G. (2020). Is the outcome after hepatectomy for transitional hepatocholangiocarcinoma different from that of hepatocellular carcinoma and mass-forming cholangiocarcinoma? A case-matched analysis. Updates Surg..

[33] Bilreiro C., Soler J.C., Ayuso J.R., Caseiro-Alves F., Ayuso C. (2021). Diagnostic value of morphological enhancement patterns in the hepatobiliary phase of gadoxetic acid-enhanced MRI to distinguish focal nodular hyperplasia from hepatocellular adenoma. Radiol. Med..

[34] Orsatti G., Zucchetta P., Varotto A., Crimì F., Weber M., Cecchin D., Bisogno G., Spimpolo A., Giraudo C., Stramare R. (2021). Volumetric histograms-based analysis of apparent diffusion coefficients and standard uptake values for the assessment of pediatric sarcoma at staging: Preliminary results of a PET/MRI study. Radiol. Med..

[35] Petralia G., Summers P.E., Agostini A., Ambrosini R., Cianci R., Cristel G., Calistri L., Colagrande S. (2020). Dynamic contrast-enhanced MRI in oncology: How we do it. Radiol. Med..

[36] Okuda K. (2002). Natural history of hepatocellular carcinoma including fibrolamellar and hepato-cholangiocarcinoma variants. J. Gastroenterol. Hepatol..

[37] Kim M., Hwang S., Ahn C.S., Kim K.H., Moon D.B., Ha T.Y., Song G.W., Jung D.H., Park G.C., Hong S.M. (2021). Postresection prognosis of combined hepatocellular carcinoma-cholangiocarcinoma according to the 2010 World Health Organization classification: Single-center experience of 168 patients. Ann. Surg. Treat. Res..

[38] Huang D.Q., El-Serag H.B., Loomba R. (2021). Global epidemiology of NAFLD-related HCC: Trends, predictions, risk factors and prevention. Nat. Rev. Gastroenterol. Hepatol..

[39] Portolani N., Baiocchi G.L., Coniglio A., Piardi T., Grazioli L., Benetti A., Ferrari Bravo A., Giulini S.M. (2008). Intrahepatic cholangiocarcinoma and combined hepatocellular-cholangiocarcinoma: A Western experience. Ann. Surg. Oncol..

[40] Park S.E., Lee S.H., Yang J.D., Hwang H.P., Hwang S.E., Yu H.C., Moon W.S., Cho B.H. (2013). Clinicopathological characteristics and prognostic factors in combined hepatocellular carcinoma and cholangiocarcinoma. Korean J. Hepato-Biliary-Pancreat. Surg..

[41] Zhou Y.-M., Zhang X.-F., Wu L.-P., Sui C.-J., Yang J.-M. (2014). Risk factors for combined hepatocellular-cholangiocarcinoma: A hospital-based case-control study. World J. Gastroenterol..

[42] Kim S., Park Y.N., Lim J., Choi G.H., Choi J., Kim K. (2014). Characteristics of combined hepatocelluar-cholangiocarcinoma and comparison with intrahepatic cholangiocarcinoma. Eur. J. Surg. Oncol..

[43] Panjala C., Senecal D.L., Bridges M.D., Kim G.P., Nakhleh R.E., Nguyen J.H.H., Harnois D.M. (2010). The Diagnostic Conundrum and Liver Transplantation Outcome for Combined Hepatocellular-Cholangiocarcinoma. Am. J. Transplant..

[44] Zuo H.-Q., Yan L.-N., Zeng Y., Yang J.-Y., Luo H.-Z., Liu J.-W., Zhou L.-X. (2007). Clinicopathological characteristics of 15 patients with combined hepatocellular carcinoma and cholangiocarcinoma. Hepatobiliary Pancreat. Dis. Int..

[45] Maximin S., Ganeshan D.M., Shanbhogue A.K., Dighe M.K., Yeh M.M., Kolokythas O., Bhargava P., Lalwani N. (2014). Current update on combined hepatocellular-cholangiocarcinoma. Eur. J. Radiol. Open.

[46] Ayas M.F., Affas S., Ayas Z., Chand M., Hadid T. (2021). Primary Combined Hepatocellular-Cholangiocarcinoma: A Case of Underdiagnosed Primary Liver Cancer. Cureus..

[47] Lee S.D., Park S.-J., Han S.-S., Kim S.H., Kim Y.-K., Lee S.-A., Ko Y.H., Hong E.K. (2014). Clinicopathological features and prognosis of combined hepatocellular carcinoma and cholangiocarcinoma after surgery. Hepatobiliary Pancreat. Dis. Int..

[48] Chantajitr S., Wilasrusmee C., Lertsitichai P., Phromsopha N. (2006). Combined hepatocellular and cholangiocarcinoma: Clinical features and prognostic study in a Thai population. J. Hepato-Biliary-Pancreat. Surg..

[49] Zhou Y.W., Li Q.F., Chen Y.Y., Wang K., Pu D., Chen X.R., Li C.H., Jiang L., Wang Y., Li Q. (2022). Clinicopathologic features, treatment, survival, and prognostic factors of combined hepatocellular and cholangiocarcinoma: A nomogram development based on SEER database and validation in multicenter study. Eur. J. Surg. Oncol..

[50] Tang D., Nagano H., Nakamura M., Wada H., Marubashi S., Miyamoto A., Takeda Y., Umeshita K., Dono K., Monden M. (2006). Clinical and pathological features of Allen’s type C classification of resected combined hepatocellular and cholangiocarcinoma: A comparative study with hepatocellular carcinoma and cholangiocellular carcinoma. J. Gastrointest. Surg..

[51] Brunt E., Aishima S., Clavien P.A., Fowler K., Goodman Z., Gores G., Gouw A., Kagen A., Klimstra D., Komuta M. (2018). cHCC-CCA: Consensus terminology for primary liver carcinomas with both hepatocytic and cholangiocytic differentiation. Hepatology.

[52] Sempoux C., Kakar S., Kondo F., Schirmacher P., Arends M.J., Fukuyama M., Klimstra D.S. (2019). Combined hepatocellular-cholangiocarcinoma and undifferentiated primary liver carcinoma. WHO Classification of Tumours: Digestive System Tumours.

[53] Gigante E., Ronot M., Bertin C., Ciolina M., Bouattour M., Dondero F., Cauchy F., Soubrane O., Vilgrain V., Paradis V. (2019). Combining imaging and tumour biopsy improves the diagnosis of combined hepatocellular-cholangiocarcinoma. Liver Int..

[54] Albano D., Stecco A., Micci G., Sconfienza L.M., Colagrande S., Reginelli A., Grassi R., Carriero A., Midiri M., Lagalla R. (2021). Whole-body magnetic resonance imaging (WB-MRI) in oncology: An Italian survey. Radiol. Med..

[55] Granata V., Grassi R., Fusco R., Belli A., Cutolo C., Pradella S., Grazzini G., La Porta M., Brunese M.C., De Muzio F. (2021). Diagnostic evaluation and ablation treatments assessment in hepatocellular carcinoma. Infect. Agents Cancer.

[56] Granata V., Fusco R., Amato D.M., Albino V., Patrone R., Izzo F., Petrillo A. (2020). Beyond the Vascular Profile: Conventional DWI, IVIM and Kurtosis in the Assessment of Hepatocellular Carcinoma. Eur. Rev. Med. Pharmacol. Sci..

[57] Granata V., Fusco R., Filice S., Catalano O., Piccirillo M., Palaia R., Izzo F., Petrillo A. (2018). The current role and future prospectives of functional parameters by diffusion weighted imaging in the assessment of histologic grade of HCC. Infect. Agents Cancer.

[58] Granata V., Fusco R., Avallone A., Catalano O., Filice F., Leongito M., Palaia R., Izzo F., Petrillo A. (2017). Major and ancillary magnetic resonance features of LI-RADS to assess HCC: An overview and update. Infect. Agents Cancer.

[59] Granata V., Grassi R., Fusco R., Setola S., Belli A., Piccirillo M., Pradella S., Giordano M., Cappabianca S., Brunese L. (2021). Abbreviated MRI Protocol for the Assessment of Ablated Area in HCC Patients. Int. J. Environ. Res. Public Health.

[60] Granata V., Fusco R., Maio F., Avallone A., Nasti G., Palaia R., Albino V., Grassi R., Izzo F., Petrillo A. (2019). Qualitative assessment of EOB-GD-DTPA and Gd-BT-DO3A MR contrast studies in HCC patients and colorectal liver metastases. Infect. Agents Cancer.

[61] Granata V., Fusco R., Setola S.V., Picone C., Vallone P., Belli A., Incollingo P., Albino V., Tatangelo F., Izzo F. (2019). Microvascular invasion and grading in hepatocellular carcinoma: Correlation with major and ancillary features according to LIRADS. Abdom. Radiol..

[62] Lian S., Zhang C., Chi J., Huang Y., Shi F., Xie C. (2020). Differentiation between nasopharyngeal carcinoma and lymphoma at the primary site using whole-tumor histogram analysis of apparent diffusion coefficient maps. Radiol. Med..

[63] Granata V., Fusco R., De Muzio F., Cutolo C., Setola S.V., Grassi R., Grassi F., Ottaiano A., Nasti G., Tatangelo F. (2022). Radiomics textural features by MR imaging to assess clinical outcomes following liver resection in colorectal liver metastases. Radiol. Med..

[64] Agazzi G.M., Ravanelli M., Roca E., Medicina D., Balzarini P., Pessina C., Vermi W., Berruti A., Maroldi R., Farina D. (2021). CT texture analysis for prediction of EGFR mutational status and ALK rearrangement in patients with non-small cell lung cancer. Radiol. Med..

[65] Iacobellis F., Di Serafino M., Brillantino A., Mottola A., Del Giudice S., Stavolo C., Festa P., Patlas M.N., Scaglione M., Romano L. (2021). Role of MRI in early follow-up of patients with solid organ injuries: How and why we do it?. Radiol. Med..

[66] Granata V., Petrillo M., Fusco R., Setola S.V., De Lutio Di Castelguidone E., Catalano O., Piccirillo M., Albino V., Izzo F., Petrillo A. (2013). Surveillance of HCC Patients after Liver RFA: Role of MRI with Hepatospecific Contrast versus Three-Phase CT Scan—Experience of High Volume Oncologic Institute. Gastroenterol. Res. Pract..

[67] Granata V., Fusco R., Avallone A., Filice F., Tatangelo F., Piccirillo M., Grassi R., Izzo F., Petrillo A. (2017). Critical analysis of the major and ancillary imaging features of LI-RADS on 127 proven HCCs evaluated with functional and morphological MRI: Lights and shadows. Oncotarget.

[68] Izzo F., Piccirillo M., Albino V., Palaia R., Belli A., Granata V., Setola S., Fusco R., Petrillo A., Orlando R. (2013). Prospective screening increases the detection of potentially curable hepatocellular carcinoma: Results in 8900 high-risk patients. HPB.

[69] Danti G., Flammia F., Matteuzzi B., Cozzi D., Berti V., Grazzini G., Pradella S., Recchia L., Brunese L., Miele V. (2021). Gastrointestinal neuroendocrine neoplasms (GI-NENs): Hot topics in morphological, functional, and prognostic imaging. Radiol. Med..

[70] Ria F., Samei E. (2020). Is regulatory compliance enough to ensure excellence in medicine?. Radiol. Med..

[71] The American College of Radiology Liver Reporting & Data System (LI- RADS). https://www.acr.org/Clinical-Resources/Reporting-and-Data-Systems/LI-RADS.

[72] Andrisani M.C., Vespro V., Fusco S., Palleschi A., Musso V., Esposito A., Coppola A., Spadafora P., Damarco F., Scaravilli V. (2021). Interobserver variability in the evaluation of primary graft dysfunction after lung transplantation: Impact of radiological training and analysis of discordant cases. Radiol. Med..

[73] Negroni D., Cassarà A., Trisoglio A., Soligo E., Berardo S., Carriero A., Stecco A. (2021). Learning curves in radiological reporting of whole-body MRI in plasma cell disease: A retrospective study. Radiol. Med..

[74] Zeng D., Xu M., Liang J.-Y., Cheng M.-Q., Huang H., Pan J.-M., Huang Y., Tong W.-J., Xie X.-Y., Lu M.-D. (2021). Using new criteria to improve the differentiation between HCC and non-HCC malignancies: Clinical practice and discussion in CEUS LI-RADS 2017. Radiol. Med..

[75] Pignata S., Gallo C., Daniele B., Elba S., Giorgio A., Capuano G., Adinolfi L.E., De Sio I., Izzo F., Farinati F. (2006). Characteristics at presentation and outcome of hepatocellular carcinoma (HCC) in the elderly. Crit. Rev. Oncol..

[76] Perrone F., Gallo C., Daniele B., Gaeta G., Izzo F., Capuano G., Adinolfi L., Mazzanti R., Farinati F., Elba S. (2002). Tamoxifen in the Treatment of Hepatocellular Carcinoma: 5-Year Results of the CLIP-1 Multicentre Randomised Controlled Trial. Curr. Pharm. Des..

[77] Granata V., Fusco R., Venanzio Setola S., Sandomenico F., Luisa Barretta M., Belli A., Palaia R., Tatangelo F., Grassi R., Izzo F. (2020). Major and ancillary features according to LI-RADS in the assessment of combined hepatocellular-cholangiocarcinoma. Radiol. Oncol..

[78] An C., Lee C.H., Byun J.H., Lee M.H., Jeong W.K., Choi S.H., Kim D.Y., Lim Y.-S., Kim Y.S., Kim J.H. (2019). Intraindividual Comparison between Gadoxetate-Enhanced Magnetic Resonance Imaging and Dynamic Computed Tomography for Characterizing Focal Hepatic Lesions: A Multicenter, Multireader Study. Korean J. Radiol..

[79] Kim Y.-Y., Kim M.-J., Kim E.H., Roh Y.H., An C. (2019). Hepatocellular Carcinoma versus Other Hepatic Malignancy in Cirrhosis: Performance of LI-RADS Version 2018. Radiology.

[80] Zheng W., Li Q., Zou X.-B., Wang J.-W., Han F., Li F., Huang L.-S., Li A.-H., Zhou J.-H. (2020). Evaluation of Contrast-enhanced US LI-RADS version 2017: Application on 2020 Liver Nodules in Patients with Hepatitis B Infection. Radiology.

[81] Marrero J.A., Kulik L.M., Sirlin C.B., Zhu A.X., Finn R.S., Abecassis M.M., Roberts L.R., Heimbach J.K. (2018). Diagnosis, Staging, and Management of Hepatocellular Carcinoma: 2018 Practice Guidance by the American Association for the Study of Liver Diseases. Hepatology.

[82] Agostini A., Borgheresi A., Carotti M., Ottaviani L., Badaloni M., Floridi C., Giovagnoni A. (2021). Third-generation iterative reconstruction on a dual-source, high-pitch, low-dose chest CT protocol with tin filter for spectral shaping at 100 kV: A study on a small series of COVID-19 patients. Radiol. Med..

[83] Park S.H., Kim Y.S., Choi J. (2021). Dosimetric analysis of the effects of a temporary tissue expander on the radiotherapy technique. Radiol. Med..

[84] Cozzi D., Moroni C., Cavigli E., Bindi A., Caviglioli C., Nazerian P., Vanni S., Miele V., Bartolucci M. (2021). Prognostic value of CT pulmonary angiography parameters in acute pulmonary embolism. Radiol. Med..

[85] Brizi M.G., Perillo F., Cannone F., Tuzza L., Manfredi R. (2021). The role of imaging in acute pancreatitis. Radiol. Med..

[86] Assadsangabi R., Babaei R., Songco C., Ivanovic V., Bobinski M., Chen Y.J., Nabavizadeh S.A. (2021). Multimodality oncologic evaluation of superficial neck and facial lymph nodes. Radiol. Med..

[87] Granata V., Grassi R., Fusco R., Galdiero R., Setola S.V., Palaia R., Belli A., Silvestro L., Cozzi D., Brunese L. (2021). Pancreatic cancer detection and characterization: State of the art and radiomics. Eur. Rev. Med. Pharmacol. Sci..

[88] Granata V., Fusco R., Catalano O., Setola S.V., Castelguidone E.D.L.D., Piccirillo M., Palaia R., Grassi R., Granata F., Izzo F. (2016). Multidetector computer tomography in the pancreatic adenocarcinoma assessment: An update. Infect. Agents Cancer.

[89] Bertocchi E., Barugola G., Nicosia L., Mazzola R., Ricchetti F., Dell’Abate P., Alongi F., Ruffo G. (2020). A comparative analysis between radiation dose intensification and conventional fractionation in neoadjuvant locally advanced rectal cancer: A monocentric prospective observational study. Radiol. Med..

[90] Agostini A., Floridi C., Borgheresi A., Badaloni M., Pirani P.E., Terilli F., Ottaviani L., Giovagnoni A. (2020). Proposal of a low-dose, long-pitch, dual-source chest CT protocol on third-generation dual-source CT using a tin filter for spectral shaping at 100 kVp for CoronaVirus Disease 2019 (COVID-19) patients: A feasibility study. Radiol. Med..

[91] Cicero G., Ascenti G., Albrecht M.H., Blandino A., Cavallaro M., D’Angelo T., Carerj M.L., Vogl T.J., Mazziotti S. (2020). Extra-abdominal dual-energy CT applications: A comprehensive overview. Radiol. Med..

[92] Budjan J., Schoenberg S.O., Attenberger U.I. (2017). CT und MRT der Leber: Wann, was, warum? [CT and MRI of the liver: When, what, why?]. Radiologe.

[93] Ahn S.J., Kim J.H., Lee S.M., Park S.J., Han J.K. (2018). CT reconstruction algorithms affect histogram and texture analysis: Evidence for liver parenchyma, focal solid liver lesions, and renal cysts. Eur. Radiol..

[94] Granata V., Cascella M., Fusco R., Dell’Aprovitola N., Catalano O., Filice S., Schiavone V., Izzo F., Cuomo A., Petrillo A. (2016). Immediate Adverse Reactions to Gadolinium-Based MR Contrast Media: A Retrospective Analysis on 10,608 Examinations. BioMed Res. Int..

[95] Palumbo P., Masedu F., De Cataldo C., Cannizzaro E., Bruno F., Pradella S., Arrigoni F., Valenti M., Splendiani A., Barile A. (2021). Real-world clinical validity of cardiac magnetic resonance tissue tracking in primitive hypertrophic cardiomyopathy. Radiol. Med..

[96] Napolitano M., Munari A.M., Di Leo G., Panarisi N.A.R., Zuin G., Fava G., Vecchi M., Sardanelli F., Zuccotti G.V. (2021). MR enterography grading of pediatric ileocolonic Crohn disease activity based on a single bowel segment. Radiol. Med..

[97] Santone A., Brunese M.C., Donnarumma F., Guerriero P., Mercaldo F., Reginelli A., Miele V., Giovagnoni A., Brunese L. (2021). Radiomic features for prostate cancer grade detection through formal verification. Radiol. Med..

[98] Granata V., Fusco R., Barretta M.L., Picone C., Avallone A., Belli A., Patrone R., Ferrante M., Cozzi D., Grassi R. (2021). Radiomics in hepatic metastasis by colorectal cancer. Infect. Agents Cancer.

[99] Granata V., Fusco R., Avallone A., De Stefano A., Ottaiano A., Sbordone C., Brunese L., Izzo F., Petrillo A. (2021). Radiomics-Derived Data by Contrast Enhanced Magnetic Resonance in RAS Mutations Detection in Colorectal Liver Metastases. Cancers.

[100] Granata V., Fusco R., Risi C., Ottaiano A., Avallone A., De Stefano A., Grimm R., Grassi R., Brunese L., Izzo F. (2020). Diffusion-Weighted MRI and Diffusion Kurtosis Imaging to Detect RAS Mutation in Colorectal Liver Metastasis. Cancers.

[101] Zhang A., Song J., Ma Z., Chen T. (2020). Combined dynamic contrast-enhanced magnetic resonance imaging and diffusion-weighted imaging to predict neoadjuvant chemotherapy effect in FIGO stage IB2-IIA2 cervical cancers. Radiol. Med..

[102] Crimì F., Capelli G., Spolverato G., Bao Q.R., Florio A., Rossi S.M., Cecchin D., Albertoni L., Campi C., Pucciarelli S. (2020). MRI T2-weighted sequences-based texture analysis (TA) as a predictor of response to neoadjuvant chemo-radiotherapy (nCRT) in patients with locally advanced rectal cancer (LARC). Radiol. Med..

[103] Zhang L., Kang L., Li G., Zhang X., Ren J., Shi Z., Li J., Yu S. (2020). Computed tomography-based radiomics model for discriminating the risk stratification of gastrointestinal stromal tumors. Radiol. Med..

[104] Gurgitano M., Angileri S.A., Rodà G.M., Liguori A., Pandolfi M., Ierardi A.M., Wood B.J., Carrafiello G. (2021). Interventional Radiology ex-machina: Impact of Artificial Intelligence on practice. Radiol. Med..

[105] Scapicchio C., Gabelloni M., Barucci A., Cioni D., Saba L., Neri E. (2021). A deep look into radiomics. Radiol. Med..

[106] Wei J., Jiang H., Gu D., Niu M., Fu F., Han Y., Song B., Tian J. (2020). Radiomics in liver diseases: Current progress and future opportunities. Liver Int..

[107] de la Pinta C., Castillo M.E., Collado M., Galindo-Pumariño C., Peña C. (2021). Radiogenomics: Hunting Down Liver Metastasis in Colorectal Cancer Patients. Cancers.

[108] Nardone V., Reginelli A., Grassi R., Boldrini L., Vacca G., D’Ippolito E., Annunziata S., Farchione A., Belfiore M.P., Desideri I. (2021). Delta radiomics: A systematic review. Radiol. Med..

[109] Brunese L., Brunese M.C., Carbone M., Ciccone V., Mercaldo F., Santone A. (2021). Automatic PI-RADS assignment by means of formal methods. Radiol. Med..

[110] Granata V., Fusco R., Avallone A., Cassata A., Palaia R., Delrio P., Grassi R., Tatangelo F., Grazzini G., Izzo F. (2020). Abbreviated MRI protocol for colorectal liver metastases: How the radiologist could work in pre surgical setting. PLoS ONE.

[111] Granata V., Fusco R., Setola S.V., Raso M.M., Avallone A., De Stefano A., Nasti G., Palaia R., Delrio P., Petrillo A. (2019). Liver radiologic findings of chemotherapy-induced toxicity in liver colorectal metastases patients. Eur. Rev. Med. Pharmacol. Sci..

[112] Granata V., Fusco R., Catalano O., Avallone A., Palaia R., Botti G., Tatangelo F., Granata F., Cascella M., Izzo F. (2017). Diag-nostic accuracy of magnetic resonance, computed tomography and contrast enhanced ultrasound in radiological multimo-dality assessment of peribiliary liver metastases. PLoS ONE.

[113] Granata V., Fusco R., Catalano O., Filice S., Amato D.M., Nasti G., Avallone A., Izzo F., Petrillo A. (2015). Early Assessment of Colorectal Cancer Patients with Liver Metastases Treated with Antiangiogenic Drugs: The Role of Intravoxel Incoherent Motion in Diffusion-Weighted Imaging. PLoS ONE.

[114] Zhang Y., Zhu Y., Zhang K., Liu Y., Cui J., Tao J., Wang Y., Wang S. (2020). Invasive ductal breast cancer: Preoperative predict Ki-67 index based on radiomics of ADC maps. Radiol. Med..

[115] Grassi R., Belfiore M.P., Montanelli A., Patelli G., Urraro F., Giacobbe G., Fusco R., Granata V., Petrillo A., Sacco P. (2021). COVID-19 pneumonia: Computer-aided quantification of healthy lung parenchyma, emphysema, ground glass and consolidation on chest computed tomography (CT). Radiol. Med..

[116] Cusumano D., Meijer G., Lenkowicz J., Chiloiro G., Boldrini L., Masciocchi C., Dinapoli N., Gatta R., Casà C., Damiani A. (2021). A field strength independent MR radiomics model to predict pathological complete response in locally advanced rectal cancer. Radiol. Med..

[117] Granata V., Fusco R., Sansone M., Grassi R., Maio F., Palaia R., Tatangelo F., Botti G., Grimm R., Curley S. (2020). Magnetic resonance imaging in the assessment of pancreatic cancer with quantitative parameter extraction by means of dynamic contrast-enhanced magnetic resonance imaging, diffusion kurtosis imaging and intravoxel incoherent motion diffusion-weighted imaging. Ther. Adv. Gastroenterol..

[118] Barile A. (2021). Correction to: Some thoughts and greetings from the new Editor-in-Chief. Radiol. Med..

[119] Wells M.L., Venkatesh S.K., Chandan V.S., Fidler J.L., Fletcher J.G., Johnson G., Hough D.M., Roberts L. (2015). Biphenotypic hepatic tumors: Imaging findings and review of literature. Gastrointest. Radiol..

[120] Hu H.-T., Shan Q.-Y., Chen S.-L., Li B., Feng S.-T., Xu E.-J., Li X., Long J.-Y., Xie X.-Y., Lu M.-D. (2020). CT-based radiomics for preoperative prediction of early recurrent hepatocellular carcinoma: Technical reproducibility of acquisition and scanners. Radiol. Med..

[121] Cellina M., Pirovano M., Ciocca M., Gibelli D., Floridi C., Oliva G. (2021). Radiomic analysis of the optic nerve at the first episode of acute optic neuritis: An indicator of optic nerve pathology and a predictor of visual recovery?. Radiol. Med..

[122] Arrigoni F., Bruno F., Gianneramo C., Palumbo P., Zugaro L., Zoccali C., Barile A., Masciocchi C. (2020). Evolution of the imaging features of osteoid osteoma treated with RFA or MRgFUS during a long-term follow-up: A pictorial review with clinical correlations. Radiol. Med..

[123] Koç A., Sezgin Ö.S., Kayıpmaz S. (2020). Comparing different planimetric methods on volumetric estimations by using cone beam computed tomography. Radiol. Med..

[124] van Assen M., Muscogiuri G., Caruso D., Lee S.J., Laghi A., De Cecco C.N. (2020). Artificial intelligence in cardiac radiology. Radiol. Med..

[125] Cicero G., Mazziotti S., Silipigni S., Blandino A., Cantisani V., Pergolizzi S., D’Angelo T., Stagno A., Maimone S., Squadrito G. (2021). Dual-energy CT quantification of fractional extracellular space in cirrhotic patients: Comparison between early and delayed equilibrium phases and correlation with oesophageal varices. Radiol. Med..

[126] Shetty A.S., Fowler K., Brunt E.M., Agarwal S., Narra V.R., Menias C.O. (2014). Combined hepatocellular-cholangiocarcinoma: What the radiologist needs to know about biphenotypic liver carcinoma. Gastrointest. Radiol..

[127] Potretzke T.A., Tan B.R., Doyle M.B., Brunt E.M., Heiken J.P., Fowler K.J. (2016). Imaging Features of Biphenotypic Primary Liver Carcinoma (Hepatocholangiocarcinoma) and the Potential to Mimic Hepatocellular Carcinoma: LI-RADS Analysis of CT and MRI Features in 61 Cases. Am. J. Roentgenol..

[128] Wang J., Li Z., Liao Y., Li J., Dong H., Peng H., Xu W., Fan Z., Gao F., Liu C. (2021). Prediction of Survival and Analysis of Prognostic Factors for Patients With Combined Hepatocellular Carcinoma and Cholangiocarcinoma: A Population-Based Study. Front. Oncol..

[129] Agrawal S., Belghiti J. (2011). Oncologic Resection for Malignant Tumors of the Liver. Ann. Surg..

[130] Ma K.W., Chok K.S.H. (2017). Importance of surgical margin in the outcomes of hepatocholangiocarcinoma. World J. Hepatol..

[131] Garancini M., Goffredo P., Pagni F., Romano F., Roman S., Sosa J.A., Giardini V. (2014). Combined hepatocellular-cholangiocarcinoma: A population-level analysis of an uncommon primary liver tumor. Liver Transplant..

[132] Cucchetti A., Piscaglia F., Grigioni A.D., Ravaioli M., Cescon M., Zanello M., Grazi G.L., Golfieri R., Grigioni W.F., Pinna A.D. (2010). Preoperative prediction of hepatocellular carcinoma tumour grade and micro-vascular invasion by means of artificial neural network: A pilot study. J. Hepatol..

[133] Bagante F., Spolverato G., Weiss M., Alexandrescu S., Marques H.P., Aldrighetti L., Maithel S.K., Pulitano C., Bauer T.W., Shen F. (2018). Surgical Management of Intrahepatic Cholangiocarcinoma in Patients with Cirrhosis: Impact of Lymphadenectomy on Peri-Operative Outcomes. World J. Surg..

[134] Kassahun W.T., Hauss J. (2008). Management of combined hepatocellular and cholangiocarcinoma. Int. J. Clin. Pract..

[135] Kim K.H., Lee S.G., Park E.H., Hwang S., Ahn C.S., Moon D.B., Ha T.Y., Song G.W., Jung D.H., Kim K.M. (2009). Surgical Treatments and Prognoses of Patients with Combined Hepatocellular Carcinoma and Cholangiocarcinoma. Ann. Surg. Oncol..

[136] Vauthey J.N., Pawlik T.M., Abdalla E.K., Arens J.F., Nemr R.A., Wei S.H., Kennamer D.L., Ellis L.M., Curley S.A. (2004). Is extended hepatectomy for hepatobiliary malignancy justified?. Ann. Surg..

[137] Sasaki A., Kawano K., Aramaki M., Ohno T., Tahara K., Takeuchi Y., Yoshida T., Kitano S. (2001). Clinicopathologic study of mixed hepatocellular and cholangiocellular carcinoma: Modes of spreading and choice of surgical treatment by reference to macroscopic type. J. Surg. Oncol..

[138] Ercolani G., Grazi G.L., Ravaioli M., Grigioni W.F., Cescon M., Gardini A., Del Gaudio M., Cavallari A. (2004). The role of lymphadenectomy for liver tumors: Further considerations on the appropriateness of treatment strategy. Ann. Surg..

[139] Yoh T., Cauchy F., Soubrane O. (2020). Oncological resection for liver malignancies: Can the laparoscopic approach provide benefits?. Ann. Surg..

[140] Patrone R., Izzo F., Palaia R., Granata V., Nasti G., Ottaiano A., Pasta G., Belli A. (2021). Minimally invasive surgical treatment of intrahepatic cholangiocarcinoma: A systematic review. World J. Gastrointest. Oncol..

[141] Vilchez V., Shah M.B., Daily M.F., Pena L., Tzeng C.W., Davenport D., Hosein P.J., Gedaly R., Maynard E. (2016). Long-term outcome of patients undergoing liver transplantation for mixed hepatocellular carcinoma and cholangiocarcinoma: An analysis of the UNOS database. HPB.

[142] Sapisochin G., de Lope C.R., Gastaca M., de Urbina J.O., López-Andujar R., Palacios F., Ramos E., Fabregat J., Castroagudín J.F., Varo E. (2014). Intrahepatic cholangiocarcinoma or mixed hepatocellular-cholangiocarcinoma in patients undergoing liver transplantation: A Spanish matched cohort multicenter study. Ann. Surg..

[143] Lee H., Ross J.S. (2017). The potential role of comprehensive genomic profiling to guide targeted therapy for patients with biliary cancer. Ther. Adv. Gastroenterol..

[144] Gentile D., Donadon M., Lleo A., Aghemo A., Roncalli M., Di Tommaso L., Torzilli G. (2019). Surgical Treatment of Hepatocholangiocarcinoma: A Systematic Review. Liver Cancer.

[145] Lunsford K., Court C., Lee Y.S., Lu D.S., Naini B.V., Harlander-Locke M.P., Busuttil R.W., Agopian V.G. (2018). Propensity-Matched Analysis of Patients with Mixed Hepatocellular-Cholangiocarcinoma and Hepatocellular Carcinoma Undergoing Liver Transplantation. Liver Transpl..

[146] Kim J.H., Yoon H.-K., Ko G.-Y., Gwon D.I., Jang C.S., Song H.-Y., Shin J.H., Sung K.-B. (2010). Nonresectable Combined Hepatocellular Carcinoma and Cholangiocarcinoma: Analysis of the Response and Prognostic Factors after Transcatheter Arterial Chemoembolization. Radiology.

[147] Yoon Y.-I., Hwang S., Lee Y.-J., Kim K.-H., Ahn C.-S., Moon D.-B., Ha T., Song G., Jung D., Lee J. (2016). Postresection outcomes of combined hepatocellular carcinomacholangiocarcinoma, hepatocellular carcinoma and intrahepatic cholangiocarcinoma. J. Gastrointest. Surg..

[148] Na S.K., Choi G.H., Lee H.C., Shin Y.M., An J., Lee D., Shim J.H., Kim K.M., Lim Y.-S., Chung Y.-H. (2018). The effectiveness of transarterial chemoembolization in recurrent hepatocellular-cholangiocarcinoma after resection. PLoS ONE.

[149] Edeline J., Touchefeu Y., Guiu B., Farge O., Tougeron D., Baumgaertner I., Ayav A., Gimenez B., Beuzit L., Pracht M. (2019). Radioembolization plus chemotherapy for first-line treatment of locally advanced intrahepatic cholangiocarcinoma: A phase 2 clinical trial. JAMA Oncol..

[150] Malone C.D., Gibby W., Tsai R., Kim S.K., Lancia S., Akinwande O., Ramaswamy R.S. (2020). Outcomes of Yttrium-90 Radioembolization for Unresectable Combined Biphenotypic Hepatocellular-Cholangiocarcinoma. J. Vasc. Interv. Radiol..

[151] Llovet J.M., Ricci S., Mazzaferro V., Hilgard P., Gane E., Blanc J.-F., De Oliveira A.C., Santoro A., Raoul J.-L., Forner A. (2008). Sorafenib in Advanced Hepatocellular Carcinoma. N. Engl. J. Med..

[152] Rizvi S., Khan S.A., Hallemeier C.L., Kelley R.K., Gores G.J. (2018). Cholangiocarcinoma—Evolving concepts and therapeutic strategies. Nat. Rev. Clin. Oncol..

[153] Kobayashi S., Terashima T., Shiba S., Yoshida Y., Yamada I., Iwadou S., Horiguchi S., Takahashi H., Suzuki E., Moriguchi M. (2018). Multicenter retrospective analysis of systemic chemotherapy for unresectable combined hepatocellular and cholangiocarcinoma. Cancer Sci..

[154] Salimon M., Prieux-Klotz C., Tougeron D., Hautefeuille V., Caulet M., Gournay J., Matysiak-Budnik T., Bennouna J., Meyo M.T., Lecomte T. (2018). Gemcitabine plus platinum-based chemotherapy for first-line treatment of hepatocholangiocarcinoma: An AGEO French multicentre retrospective study. Br. J. Cancer.

[155] Trikalinos N.A., Zhou A., Doyle M.B.M., Fowler K.J., Morton A., Vachharajani N., Amin M., Keller J.W., Chapman W.C., Brunt E.M. (2018). Systemic Therapy for Combined Hepatocellular-Cholangiocarcinoma: A Single-Institution Experience. J. Natl. Compr. Cancer Netw..

[156] Kim E.J., Yoo C., Kang H.J., Kim K., Ryu M., Park S.R., Lee D., Choi J., Shim J.H., Kim K.M. (2021). Clinical outcomes of systemic therapy in patients with unresectable or metastatic combined hepatocellular-cholangiocarcinoma. Liver Int..

[157] Arai Y., Totoki Y., Hosoda F., Shirota T., Hama N., Nakamura H., Ojima H., Furuta K., Shimada K., Okusaka T. (2013). Fibroblast growth factor receptor 2 tyrosine kinase fusions define a unique molecular subtype of cholangiocarcinoma. Hepatology.

[158] Abou-Alfa G.K., Sahai V., Hollebecque A., Vaccaro G., Melisi D., Al-Rajabi R., Paulson A.S., Borad M.J., Gallinson D., Murphy A.G. (2020). Pemigatinib for previously treated, locally advanced or metastatic cholangiocarcinoma: A multicentre, open-label, phase 2 study. Lancet Oncol..

[159] Bekaii-Saab T., Bridgewater J., Normanno N. (2021). Practical considerations in screening for genetic alterations in cholangiocarcinoma. Ann. Oncol..

